# Sulbactam-durlobactam susceptibility test method development and quality control ranges for MIC and disk diffusion tests

**DOI:** 10.1128/jcm.01228-23

**Published:** 2023-12-14

**Authors:** Sarah M. McLeod, Nicole M. Carter, Michael D. Huband, Maria M. Traczewski, Patricia A. Bradford, Alita A. Miller

**Affiliations:** 1 Entasis Therapeutics Inc. (an affiliate of Innoviva Specialty Therapeutics, Inc.), Waltham, Massachusetts, USA; 2 JMI Labs, North Liberty, Iowa, USA; 3 Clinical Microbiology Institute, Wilsonville, Oregon, USA; 4 Antimicrobial Development Specialists, LLC, Nyack, New York, USA; Medical College of Wisconsin, Milwaukee, Wisconsin, USA

**Keywords:** sulbactam-durlobactam, susceptibility testing, quality control

## Abstract

Sulbactam-durlobactam is a β-lactam/β-lactamase inhibitor combination developed to treat hospital-acquired and ventilator-associated bacterial pneumonia caused by *Acinetobacter baumannii-calcoaceticus* complex (ABC). Durlobactam is a diazabicyclooctane β-lactamase inhibitor with potent activity against Ambler classes A, C, and D serine β-lactamases and restores sulbactam activity against multidrug-resistant ABC. Studies were conducted to establish sulbactam-durlobactam antimicrobial susceptibility testing methods for both broth microdilution minimal inhibitory concentration (MIC) and disk diffusion tests as well as quality control (QC) ranges. To establish the MIC test method, combinations of sulbactam and durlobactam were evaluated using a panel of genetically characterized *A. baumannii* isolates which were categorized as predicted to be susceptible or resistant based on the spectrum of β-lactamase inhibition by durlobactam. MIC testing with doubling dilutions of sulbactam with a fixed concentration of 4 µg/mL of durlobactam resulted in the greatest discrimination of the pre-defined susceptible and resistant strains. Similarly, the sulbactam/durlobactam 10/10 µg disk concentration showed the best discrimination as well as correlation with the MIC test. *A. baumannii* NCTC 13304 was selected for QC purposes because it assesses the activity of both sulbactam and durlobactam with clear endpoints. Multi-laboratory QC studies were conducted according to CLSI M23 Tier 2 criteria. A sulbactam-durlobactam broth MIC QC range of 0.5/4–2/4 µg/mL and a zone diameter QC range of 24–30 mm were determined for *A. baumannii* NCTC 13304 and have been approved by CLSI. These studies will enable clinical laboratories to perform susceptibility tests with accurate and reproducible methods.

## INTRODUCTION

Sulbactam-durlobactam is a β-lactam/β-lactamase inhibitor combination (BL/BLI) approved by the US FDA (May 2023) for the treatment of patients 18 years of age and older with hospital-acquired bacterial pneumonia and ventilator-associated bacterial pneumonia caused by susceptible isolates of *Acinetobacter baumannii-calcoaceticus* complex (ABC) (1). Sulbactam is a penicillin derivative β-lactamase inhibitor of a subset of class A β-lactamases that also has intrinsic antibacterial activity against a few Gram-negative bacterial species including *Neisseria gonorrhoeae*, *Bacteroides fragilis*, and *Acinetobacter* spp. through inhibition of penicillin-binding proteins PBP1 and PBP3 (2, 3). However, degradation of sulbactam by a variety of β-lactamases present in most clinical ABC isolates limits its clinical use (4–6). Durlobactam is a diazabicyclooctane (DBO) β-lactamase inhibitor with an expanded spectrum of activity compared to other DBO inhibitors, which includes coverage of a broad range of class A, C, and D serine β-lactamases (4). Durlobactam does not have clinically relevant intrinsic antibacterial activity against *A. baumannii*; however, it does have some activity against isolates of *Enterobacterales*, due to inhibition of PBP2 in these organisms (4).

For most antibacterials, including β-lactams, the establishment of antimicrobial susceptibility test methods is reasonably straightforward; the minimal inhibitory concentration (MIC) measures the bacterial response to various drug concentrations at a defined endpoint in time (7–11). Subsequently, the concentration of antibacterial used in disk diffusion tests is based on a dose-response to give measurable zones of inhibition (12). However, with β-lactam–β-lactamase inhibitor combinations, this is more complicated because the inhibitor has a different effect on various strains based on the type and amount of β-lactamase expressed in the strain. The combinations that have been approved to date have utilized different methodologies for performing MIC tests, with some using a ratio of β-lactam to β-lactamase inhibitor and some using doubling dilutions of the β-lactam with a fixed concentration of inhibitor. Early combinations used the ratio to mimic the dose that was given to patients in the human formulation (13, 14). Later, the fixed concentration of inhibitor in MIC tests was determined to aid in the identification of resistant strains (15). The amount of inhibitor to use in disk tests has also varied in both the amount and rationale (16–18). The objective of this study was to determine the best test methodologies for MIC and disk diffusion testing of sulbactam-durlobactam, and then establish quality control (QC) ranges to use with those tests.

## MATERIALS AND METHODS

### Antibiotics and media

Durlobactam was synthesized by Entasis Therapeutics Inc., an affiliate of Innoviva Specialty Therapeutics, Inc. Sulbactam, ampicillin, minocycline, meropenem, and imipenem were obtained from U.S. Pharmacopeia (USP). For the MIC quality control study, media lots of cation-adjusted Mueller-Hinton broth were obtained from Becton Dickinson (Lot# 5257869), Difco (Lot# 4045151), and Oxoid (Lot# 1743805). For the disk quality control study, media lots of Mueller-Hinton agar were obtained from ThermoFisher Scientific (Remel, Lot# 191341), Becton Dickinson (BBL, Lot# 7173648), and Hardy Diagnostics (Lot# 17212). For the disk quality control study, sulbactam-durlobactam 10/10 µg disks were manufactured by ThermoFisher Scientific (Basingstoke, UK) and Mast (Merseyside, UK). Ampicillin-sulbactam 10/10 µg and meropenem 10 µg disks were obtained from Becton Dickinson.

### Bacterial strains

The bacterial strains used for the MIC methods study were part of the microbiological culture collection housed at Entasis Therapeutics Inc., Waltham, MA, USA (labeled “ARC”). Isolates labeled “CDC” were obtained from the US Centers for Disease Control and Prevention (CDC) and FDA Antimicrobial Resistance (AR) Isolate Bank (https://www.cdc.gov/drugresistance/resistance-bank/index.html) (*A. baumannii* panel). β-lactamase genes and PBP3 alleles had been previously characterized by whole genome sequencing (Table S1). For the study to determine the disk concentration, the first set of experiments with five different disk concentrations was conducted with 58 isolates of ABC that demonstrate a spectrum of sulbactam-durlobactam MIC values and were selected from the collection at JMI Laboratories (North Liberty, IA, USA). The second experiment focused on two disk concentrations and utilized 260 clinical isolates of ABC obtained during worldwide surveillance studies during 2013–2015 plus 40 ABC isolates with high sulbactam-durlobactam MIC values. For the quality control studies, the following strains were used: *Escherichia coli* ATCC 25922, *Klebsiella pneumoniae* ATCC 700603, *A. baumannii* ARC3492, *A. baumannii* NCTC 13304, and *E. coli* NCTC 13353.

### Broth MIC testing method development

The MIC for each organism was determined by broth microdilution using the Clinical and Laboratory Standards Institute (CLSI) M07 guideline (19). For the MIC methods study, sulbactam was tested using serial twofold drug dilutions. Durlobactam was added in a fixed ratio of 1:1, 2:1, 4:1, or 8:1 sulbactam to durlobactam or at fixed concentrations of 0.5, 1, 2, 4, or 8 µg/mL. Prior to testing, the susceptibility to sulbactam-durlobactam was predicted for 91 strains of *A. baumannii*, based on whole genome sequence analysis, and the panel of isolates was prespecified as “predicted inhibited” or “predicted resistant.” Histogram plots were used to analyze the performance of the different testing conditions and the ability to distinguish isolates that were predicted to not be inhibited by durlobactam. Following the determination of the optimal MIC method, the MIC values of sulbactam-durlobactam were performed using twofold dilutions of sulbactam in the presence of a fixed concentration of 4 µg/mL durlobactam for all subsequent studies.

### Disk diffusion evaluation studies

To determine the optimal disk concentration, sulbactam-durlobactam disks were prepared at JMI Laboratories. Disk concentrations studied included (sulbactam/durlobactam in micrograms) 10/10, 10/5, 7.5/7.5, 5/10, and 5/5 µg. Disk diffusion testing was performed in duplicate and according to CLSI M02 guidelines (20). Any zone diameters with a variation >3 mm between duplicates were retested. Disk zone and MIC values that resulted in major or very major errors, two dilutions above or below the intermediate MIC value, were repeated by broth microdilution and disk diffusion methods. If the error remained, the first value was used. If the retest eliminated the error, the test was performed a third time, and the result for two of three tests was used. Measured zone diameters and MIC values were used to generate scattergram analyses according to CLSI guidelines for test development (21, 22). Error rates were based on MIC breakpoints of ≤4/4 µg/mL for susceptible, 8/4 µg/mL for intermediate, and ≥16/4 µg/mL for resistant. The zone diameter breakpoints for each disk were drawn to minimize the errors.

### Establishment of quality control ranges

For the establishment of quality control ranges, CLSI M23 Tier 2 multi-laboratory studies were conducted in eight independent laboratories for both MIC and disk tests according to CLSI guidelines (21). Tests were performed in 10 replicates on at least three different days with no more than four replicates on any given day. Colony counts (expressed as colony-forming units per milliliter) were performed to verify the starting inoculum concentrations (minimum of five inoculum verifications per organism per participating laboratory). Cefepime and cefepime-tazobactam, which are a BL and BL/BLI combination similar to sulbactam and sulbactam-durlobactam, were used as control drugs. For the quality control disk diffusion testing, sulbactam/durlobactam (10/10 µg) disks were commercially prepared by two different manufacturers. Ampicillin-sulbactam 10/10 µg and meropenem 10 µg disks were used for controls. Quality control ranges for each reference strain were calculated using the CLSI method or RangeFinder statistical program for MIC testing and the Gavan statistic or RangeFinder statistical program for disk diffusion testing (21, 23, 24).

## RESULTS AND DISCUSSION

### Broth MIC test method development for sulbactam-durlobactam

When determining the *in vitro* susceptibility of bacterial strains to BL/BLI combinations, there are two methods by which the relative concentrations of each component of the combination can be varied. In one method, both compounds are simultaneously serially diluted in a fixed ratio. For example, both amoxicillin-clavulanate and ampicillin-sulbactam are tested at a 2:1 ratio of BL to BLI. The rationale for this testing paradigm was that this ratio mimics the ratio of both components in the clinical formulation that is given to patients. For ampicillin-sulbactam, there was very little differentiation between various ratios or concentrations used in MIC testing (25–27).

Other BL/BLI combinations, including piperacillin-tazobactam, ceftazidime-avibactam, imipenem-relebactam, and meropenem-vaborbactam, use fixed inhibitor concentrations of 4 or 8 µg/mL (28). For piperacillin-tazobactam and ceftazidime-avibactam, a predictor panel of isolates that produce well-characterized β-lactamases was used to assess the performance of MIC tests. This method uses the rationale that with the knowledge of the spectrum of activity of the BLI, one could reasonably predict whether an isolate will test with a low or high MIC for that BL/BLI combination. This method showed that fixed ratios of BL to BLI had a tendency to overpredict susceptibility, and that a constant concentration of 4 µg/mL most accurately separated susceptible from resistant isolates (15, 29).

The β-lactamase inhibition spectrum and potency of durlobactam have been thoroughly studied, in both enzymatic and susceptibility studies using isogenic panels expressing individual β-lactamases (4, 30). Using these data, a predictor panel of 91 *A. baumannii* isolates was constructed either from the strain collection at Entasis Therapeutics (*N* = 68) or from the CDC AR bank *A. baumannii* panel (*N* = 23). These isolates had undergone whole genome sequencing and were classified as either “predicted inhibited” (*N* = 71) or “predicted not inhibited” (*N* = 20) based on the resistance elements defined by whole genome sequencing combined with knowledge of the spectrum of inhibition of durlobactam prior to the start of testing. Of the 91 isolates tested, only 11 had an MIC of ≤4 µg/mL for sulbactam alone, which was consistent with the whole genome sequencing data showing the presence or absence of genes that encode for β-lactamases predicted to hydrolyze sulbactam in these isolates (31). Several selected isolates also encoded for PBP3 mutant alleles associated with resistance to sulbactam (3, 32, 33).

Using this “predictor” panel of 91 *A*. *baumannii* isolates, broth MIC values were determined using weight-to-weight ratios of 1:1, 2:1, and 4:1 of sulbactam to durlobactam or using sulbactam in the presence of fixed concentrations of durlobactam at 1, 2, and 4 µg/mL. The addition of durlobactam to sulbactam significantly reduced the sulbactam MIC values against most of the isolates tested (Table S1). As expected, for isolates susceptible to sulbactam alone, there was minimal change observed in the sulbactam MIC in the presence of durlobactam.

**Fig 1:**
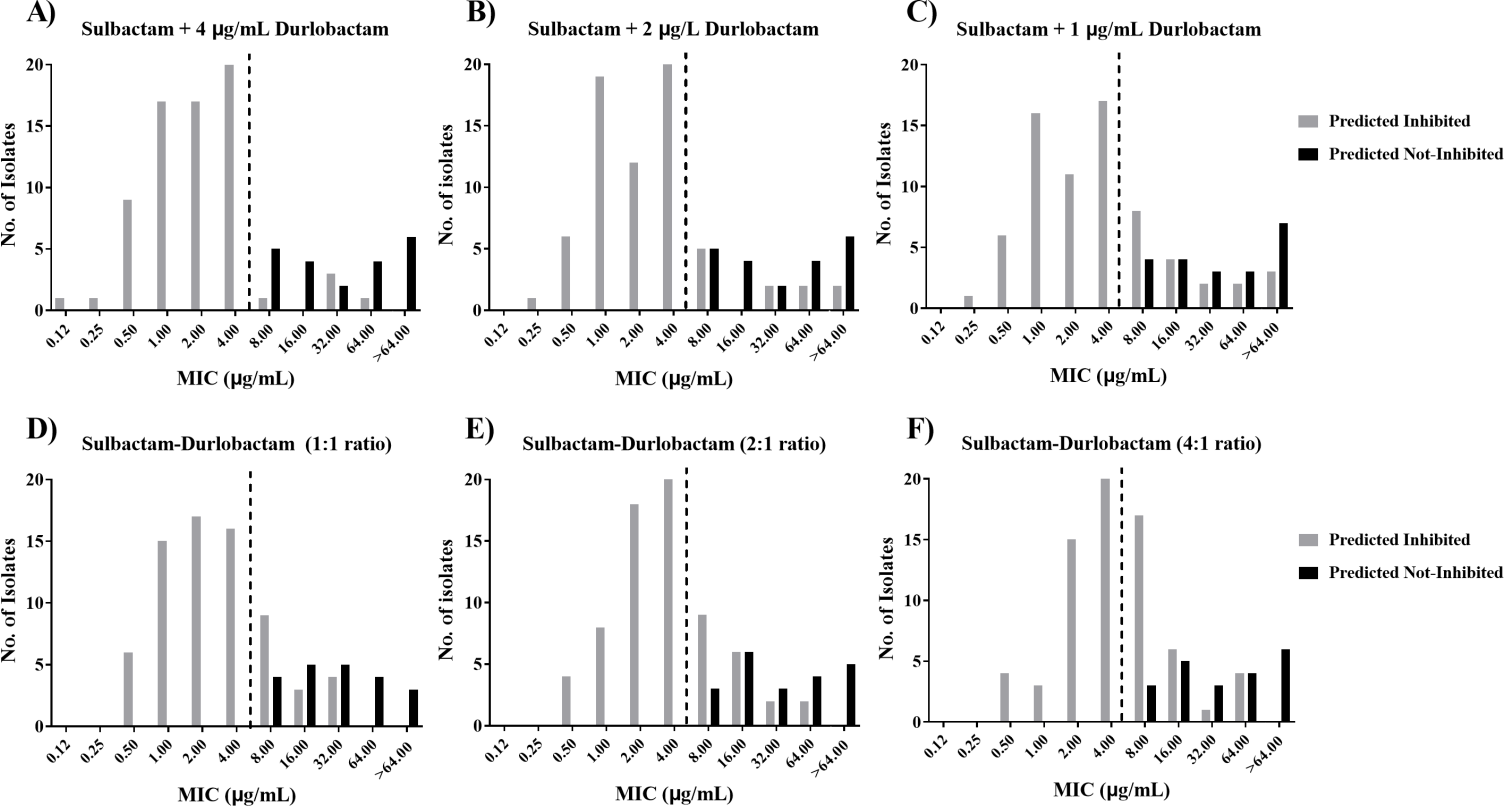
MIC distributions of sulbactam combined with durlobactam at various ratios (4:1, 2:1, or 1:1) or fixed concentrations (4, 2, or 1 µg/mL). Histograms depicting the MIC distribution of sulbactam combined with durlobactam at various ratios or concentrations against a panel of 91 clinical isolates. Isolates were defined as having β-lactamases or PBP3 alleles that should be inhibited (gray bars) or not inhibited (black bars) by sulbactam-durlobactam based on the results of whole-genome sequencing and the spectrum of inhibition of durlobactam or sulbactam against these enzymes. The sulbactam-durlobactam breakpoint of 4/4 µg/mL is depicted with a black dotted line. (**A**) Sulbactam tested with a fixed concentration of 4 µg/mL durlobactam. (**B**) Sulbactam tested with a fixed concentration of 2 µg/mL durlobactam. (**C**) Sulbactam tested with a fixed durlobactam concentration of 1 µg/mL. (**D**) Sulbactam and durlobactam tested in a 1:1 ratio. (**E**) Sulbactam and durlobactam tested in a 2:1 ratio. (**F**) Sulbactam and durlobactam tested in a 4:1 ratio.

Selection of the optimal combination was based on whether the sulbactam-durlobactam combination being tested effectively distinguished isolates predicted to be inhibited by sulbactam-durlobactam and/or PBP3 mutations found in the isolate. Comparisons of histograms of the MIC distributions showed that titrating sulbactam in the presence of a fixed concentration of 4 µg/mL durlobactam most accurately separated the “predicted inhibited” from the “predicted not inhibited” isolates (Fig. 1). Using sulbactam in the presence of a fixed concentration of 4 µg/mL durlobactam, there were five isolates that were predicted to be inhibited by sulbactam-durlobactam with MIC values above the breakpoint (Table S1). Titration of ratios of sulbactam-durlobactam or titrating sulbactam in the presence of <4 µg/mL durlobactam resulted in a trend toward underpredicted susceptibility of some clinical isolates. Compared to sulbactam tested with a fixed durlobactam concentration of 4 µg/mL, testing of sulbactam with fixed durlobactam concentrations of 2 or 1 µg/mL resulted in an additional 5 and 13 “predicted inhibited” isolates, respectively, that tested with MIC values above the susceptible breakpoint. Similarly, compared to sulbactam with durlobactam fixed at 4 µg/mL, sulbactam:durlobactam ratios of 1:1, 2:1, and 4:1 resulted in an additional 10, 13, and 22 “predicted inhibited” isolates, respectively, that tested with MIC values above the breakpoint. In these cases, larger numbers of “predicted inhibited” strains tested as resistant, suggesting that insufficient β-lactamase inhibition by durlobactam *in vitro* had occurred at these concentrations to fully restore sulbactam to wild-type activity levels.

**Fig 2:**
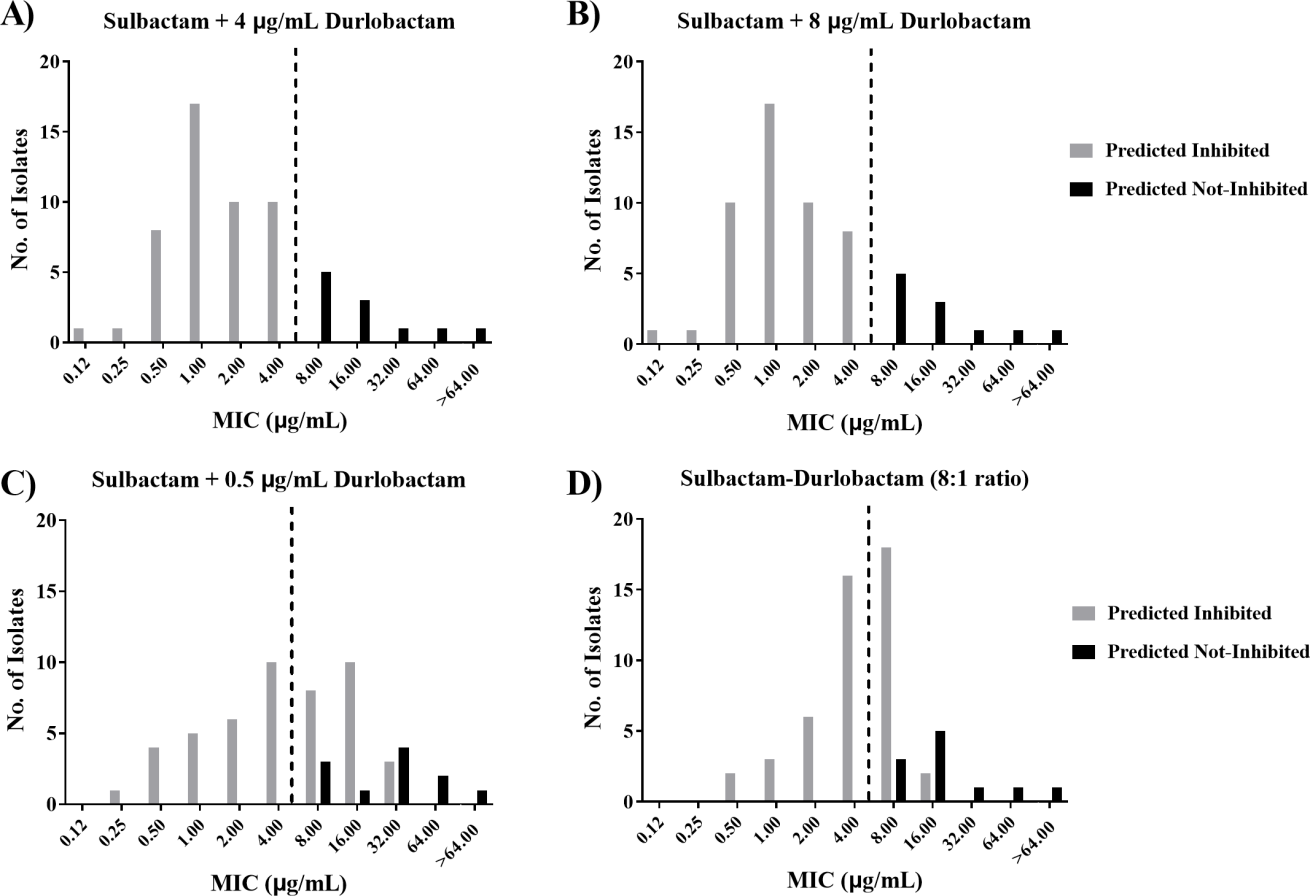
MIC distributions of sulbactam combined with durlobactam at various concentrations (4, 8, or 0.5 µg/mL) or an 8:1 ratio. MIC distributions of sulbactam combined with durlobactam at various concentrations or an 8:1 ratio against a panel of 58 clinical isolates. Isolates were defined as having β-lactamases or PBP3 alleles that should be inhibited (gray bars) or not inhibited (black bars) by sulbactam-durlobactam based on the results of whole-genome sequencing and the spectrum of inhibition of durlobactam or sulbactam against these enzymes. The sulbactam-durlobactam breakpoint of 4/4 µg/mL is depicted with a black dotted line. (A) Sulbactam tested with a fixed concentration of 4 ug/mL durlobactam. (B) Sulbactam tested with a fixed concentration of 8 ug/mL durlobactam. (C) Sulbactam tested with a fixed concentration of 0.5 ug/mL durlobactam. (D) Sulbactam and durlobactam tested in a 8:1 ratio.

To rule out the possibility that higher or lower amounts of durlobactam, such as a fixed concentration of 8 or 0.5 µg/mL durlobactam, or other testing paradigms such as an 8:1 ratio would be more beneficial than a titration of sulbactam in the presence of 4 µg/mL durlobactam, a smaller subset of 58 isolates was profiled in a similar manner applied to the set of 91 isolates. Four different testing paradigms were used: fixed concentrations of 0.5, 4, and 8 µg/mL durlobactam and an 8:1 sulbactam-durlobactam ratio. MIC distributions showed very little difference between the fixed durlobactam concentrations of 4 and 8 µg/mL, while the 0.5 µg/mL durlobactam concentration and the 8:1 ratio had many more isolates that were predicted to be inhibited but tested as above the preliminary breakpoint (Fig. 2). Because there was no benefit of increasing durlobactam to 8 µg/mL, the susceptibility testing paradigm deemed optimal was to hold the concentration of durlobactam constant at 4 µg/mL while varying the sulbactam concentration in twofold increments. This testing paradigm for susceptibility was in good agreement with the pharmacokinetic/pharmacodynamic (PK/PD) targets determined within *in vitro* and *in vivo* infection model systems where both sulbactam and durlobactam exposure targets are normalized by the potentiated sulbactam-durlobactam MIC (MIC of sulbactam in the presence of 4 µg/mL of durlobactam) (34). In these studies, %*f*T > MIC and *f*AUC_0–24_/MIC were determined to be the PK/PD drivers for sulbactam and durlobactam, respectively. Within a sulbactam-durlobactam MIC ≤4/4 µg/mL, the sulbactam bactericidal activity was restored when durlobactam *f*AUC_0–24_/MIC = 10 and sulbactam concentrations exceeded the MIC for 50% of the dosing interval. This relationship was the same for isolates that were evaluated across an MIC range from 0.5/4 to 4/4 µg/mL (34).

### Disk diffusion test method development for sulbactam-durlobactam

Development of the disk diffusion test method for sulbactam-durlobactam was conducted prior to the availability of the joint CLSI-EUCAST procedure for optimizing disk contents (22, 35). Therefore, as a first step to determine the optimal concentrations of sulbactam and durlobactam for disk diffusion testing, five different disk potencies (10/10, 10/5, 7.5/7.5, 5/10, and 5/5 sulbactam/durlobactam in micrograms) were tested simultaneously with broth microdilution MICs of sulbactam-durlobactam against a small panel of *A. baumannii* isolates (*n* = 58) that represented a range of sulbactam-durlobactam MIC values (≤0.015/4 to >32/4 µg/mL). These results were plotted in scattergrams and are shown in Fig. S1 to S5. The sulbactam/durlobactam 10/10 µg and 10/5 µg disks delivered optimal zone diameters for susceptible MIC values as recommended by CLSI (15–35 mm) (21) (Fig. S1 and S2). Sulbactam/durlobactam 10/10 µg and 10/5 µg disks demonstrated acceptable error rates with no very major or major errors and minor error rates of <40% (33.3% and 27.8%, respectively). Based on this error-rate bounding analysis that identifies categorical differences, the sulbactam/durlobactam 10/10 µg and 10/5 µg disks breakpoints of ≥18 and ≥17 mm for susceptible and ≤13 and ≤12 mm for resistant, respectively, were chosen for further analysis.

**Fig 3:**
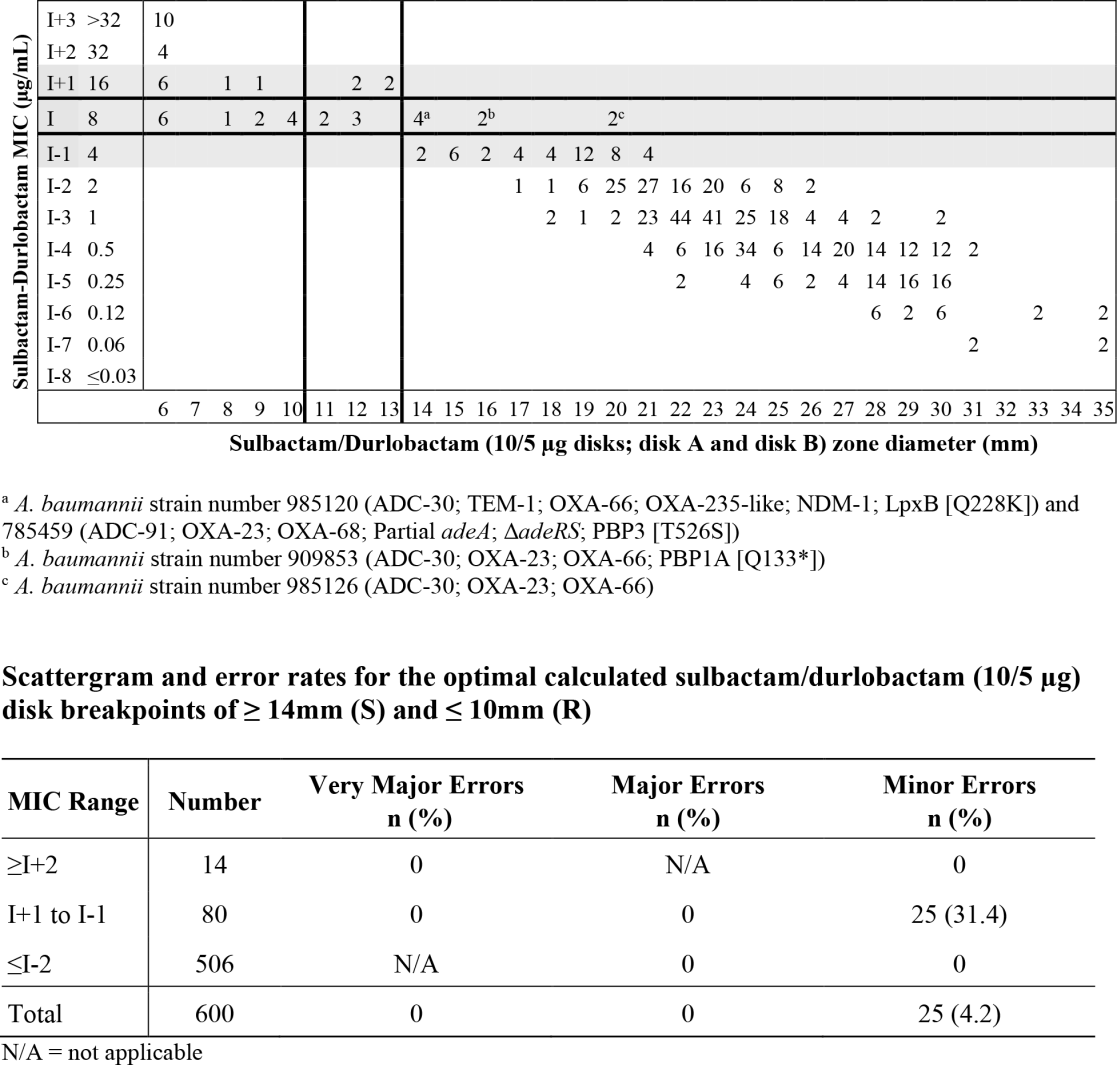
Scattergram of recommended disk breakpoints and table of error rates, based on the error-rate bounded method, for sulbactam-durlobactam (fixed 4 µg/mL) MIC values versus sulbactam-durlobactam 10/5 µg disks against 300 *A*. *baumannii-calcoaceticus* complex isolates.

The second stage of disk development examined a larger set of clinical isolates of *A. baumannii* (*n* = 300; sulbactam-durlobactam MIC range: 0.06/4 to >32/4 µg/mL) against the sulbactam/durlobactam 10/10 µg and 10/5 µg disks. MIC values of sulbactam-durlobactam were compared to zone diameter values obtained with the two disks and the results obtained were compared in scattergrams. Potential disk diffusion breakpoints were determined based on calculated scattergram error rates using the error-rate bounded method for sulbactam-durlobactam MIC values versus sulbactam-durlobactam zone diameter values for each disk concentration (21). Recommended disk diffusion breakpoints of ≥14 mm for susceptible (S) and ≤10 mm for resistant (R) for sulbactam-durlobactam 10/5 µg disks against *A. baumannii* are shown in Fig. 3. Applying these disk diffusion breakpoints resulted in acceptable error rates with no very major or major errors, a minor error rate of 31.4% (<40% required) within the MIC zone encompassing a dilution above and below the intermediate MIC value, and an overall minor error rate of 4.2%.

**Fig 4:**
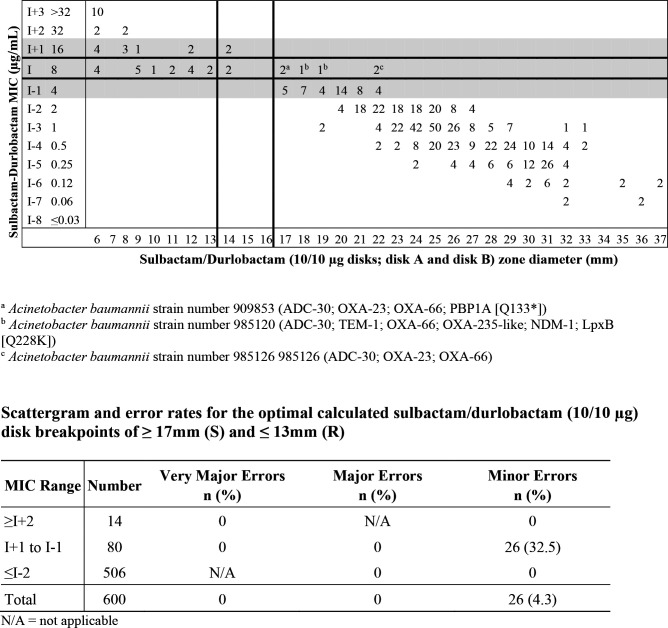
Scattergram of recommended disk breakpoints and table of error rates, based on the error-rate bounded method, for sulbactam-durlobactam (fixed 4 µg/mL) MIC values versus sulbactam-durlobactam 10/10 µg disks against 300 *A*. *baumannii-calcoaceticus* complex isolates.

Similarly, the FDA recommended disk diffusion breakpoints of ≥17 mm for susceptible and ≤13 mm for resistant for sulbactam-durlobactam 10/10 µg disks against *A. baumannii* are shown in Fig. 4. Applying these breakpoint criteria resulted in acceptable error rates against the *A. baumannii* isolates with no very major or major errors, a minor error rate of 32.5% (<40% required) within the MIC zone encompassing a dilution above and below the intermediate MIC value, and an overall minor error rate of 4.3%. Based on CLSI error-rate bounding analysis, the sulbactam-durlobactam 10/10 µg disks delivered near optimal zone diameter breakpoints of ≥17 mm for susceptible and ≤13 mm for resistant. Therefore, the sulbactam-durlobactam 10/10 µg disk mass was selected for development due to the ability of this combination to provide greater separation of susceptible and resistant *A. baumannii* populations while minimizing the occurrence of very major, major, and minor errors.

### Quality control

Collaborative *in vitro* studies were designed to collect Tier 2 quality control data to evaluate inter- and intra-laboratory reproducibility of sulbactam-durlobactam in both MIC and disk diffusion tests as outlined in CLSI document M23 (21). With any new compound or antibacterial combination that is developed, the appropriate quality control strain(s) must be chosen. For a BL/BLI combination, the quality control strain must be able to detect both components of the combination, that is the strain must express a β-lactamase that causes resistance to the parent β-lactam, but has its susceptibility restored by the addition of the β-lactamase inhibitor. With sulbactam-durlobactam, all MIC values for *E. coli* ATCC 25922 and *K. pneumoniae* ATCC 700603 were ≤0.03/4 and ≤0.06/4 µg/mL, respectively (data not shown), due to the intrinsic activity of durlobactam against these strains of *Enterobacterales* (4). Therefore, neither of these strains is appropriate for performing the quality control with sulbactam-durlobactam. Two strains of *A. baumannii* [NCTC 13304 (ADC-30; TEM-1; OXA-23; OXA-66) and ARC3492 (ADC-52-like; TEM-1; OXA-24; OXA-132; PBP3 [Q488K])] were assessed for their suitability in performing quality control. Both strains expressed β-lactamases that cause resistance to sulbactam alone but are inhibited by durlobactam. During the multi-lab study, it was noted that *A. baumannii* ARC 3492 had some very slightly cloudy wells between wells with good growth and the first well with complete inhibition of growth, making the endpoint difficult to determine. There were no issues in reading the sulbactam-durlobactam MIC endpoints with *A. baumannii* NCTC 13304; therefore, this strain was chosen as the appropriate organism for quality control testing.

**Fig 5:**
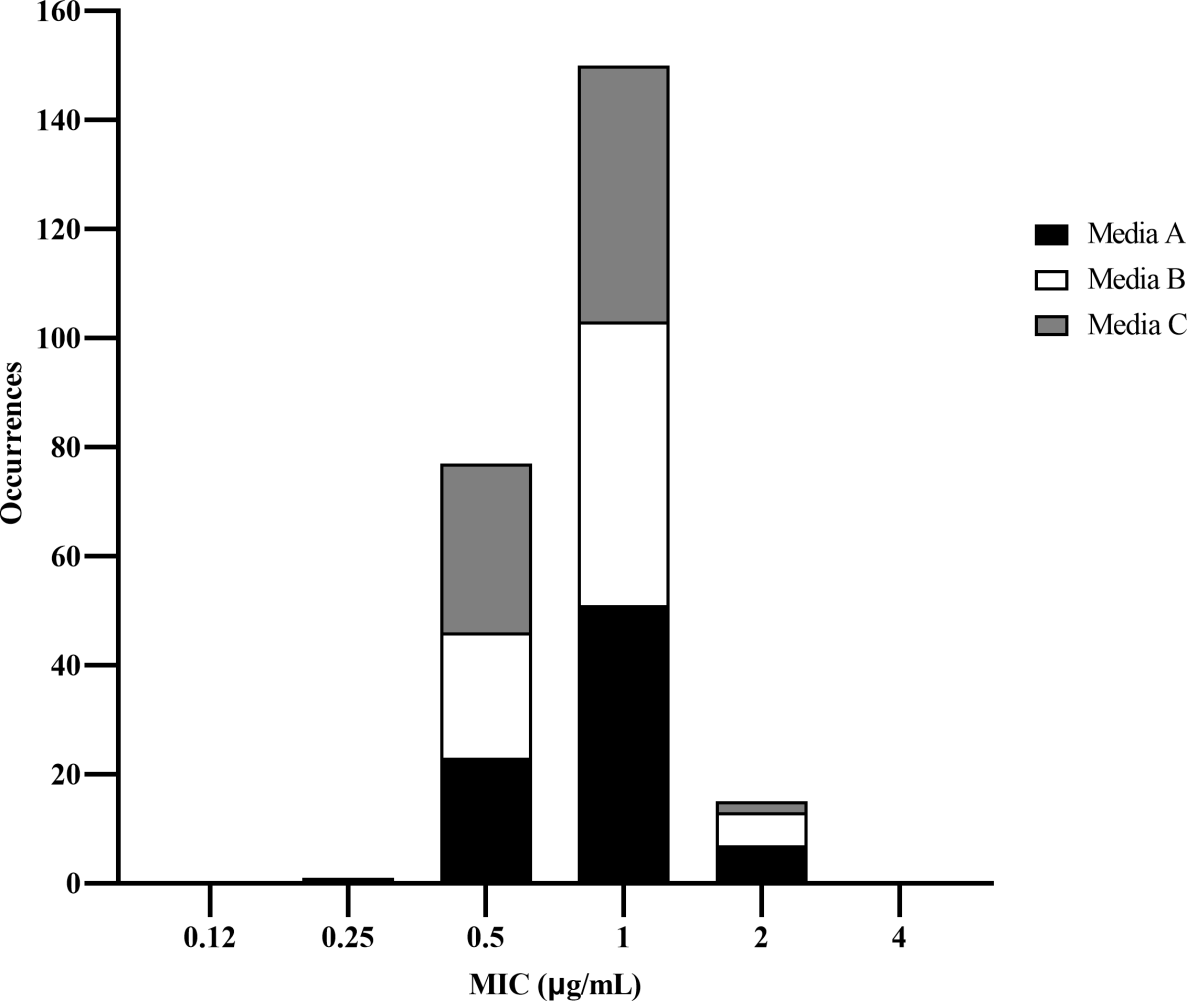
Frequency distribution of replicates of sulbactam-durlobactam MIC values with *A. baumannii* NCTC 13304 from multi-lab study by media lot.

A CLSI M23 Tier 2 multi-laboratory study was completed to establish quality control ranges for sulbactam-durlobactam broth microdilution MIC testing with doubling dilutions of sulbactam in the presence of 4 µg/mL of durlobactam. All of the replicates for control drugs cefepime and cefepime-tazobactam were within CLSI-defined quality control ranges for *Escherichia coli* ATCC 25922 and *E. coli* NCTC 13353, respectively (data not shown). The replicates of sulbactam-durlobactam MIC values generated in different media lots with *A. baumannii* NCTC 13304 are shown in Fig. 5. No bias was observed between the different media lots, and there was a strong modal MIC value at 1/4 µg/mL. Sulbactam-durlobactam MIC values (99.6%) fell within a tight 3 dilution range between 0.5/4 and 2/4 µg/mL. For *A. baumannii* NCTC 13304, sulbactam alone had an MIC range of 16–64 µg/mL while durlobactam alone had an MIC range of 32–128 µg/mL, indicating that this strain was capable of controlling for both components of the sulbactam-durlobactam combination (Table 1). These data were used by CLSI to approve a sulbactam-durlobactam broth microdilution MIC quality control range of 0.5/4−2/4 µg/mL with *A. baumannii* NCTC 13304 (Table 1) (28).

**TABLE 1:** CLSI-approved quality control ranges for susceptibility testing with sulbactam-durlobactam

Agent	Reference strain	Broth MIC		Disk diffusion (10/10 µg) sulbactam/durlobactam	
		QC range (µg/mL)	% in range	QC range (mm)	% in range
Sulbactam-durlobactam	*A. baumannii* NCTC 13304^*a*^	0.5/4–2/4	99.6	24–30	100
	*E. coli* ATCC 25922	No range approved^*b*^		26–32	99.8
	*K. pneumoniae* ATCC 700603	No range approved^*b*^			
Sulbactam	*A. baumannii* NCTC 13304	16–64	98.8		
	*E. coli* ATCC 25922	16–64	100		
	*K. pneumoniae* ATCC 700603	32–128	99.6		
Durlobactam	*A. baumannii* NCTC 13304	32–128	96.7		
	*E. coli* ATCC 25922	0.12–0.5	97.1		
	*K. pneumoniae* ATCC 700603	No range approved			

^*a*^ *A. baumannii* NCTC 13304 is recommended for routine QC to control for both components of the sulbactam-durlobactam combination.

^*b*^ No range could be set because the durlobactam MICs were <4 µg/mL for both *E. coli* ATCC 25922 and *K. pneumoniae* ATCC 700603; therefore, all results were off-scale. Reproduced from reference (27). No range could be set due to the wide distribution of MIC values.

**Fig 6:**
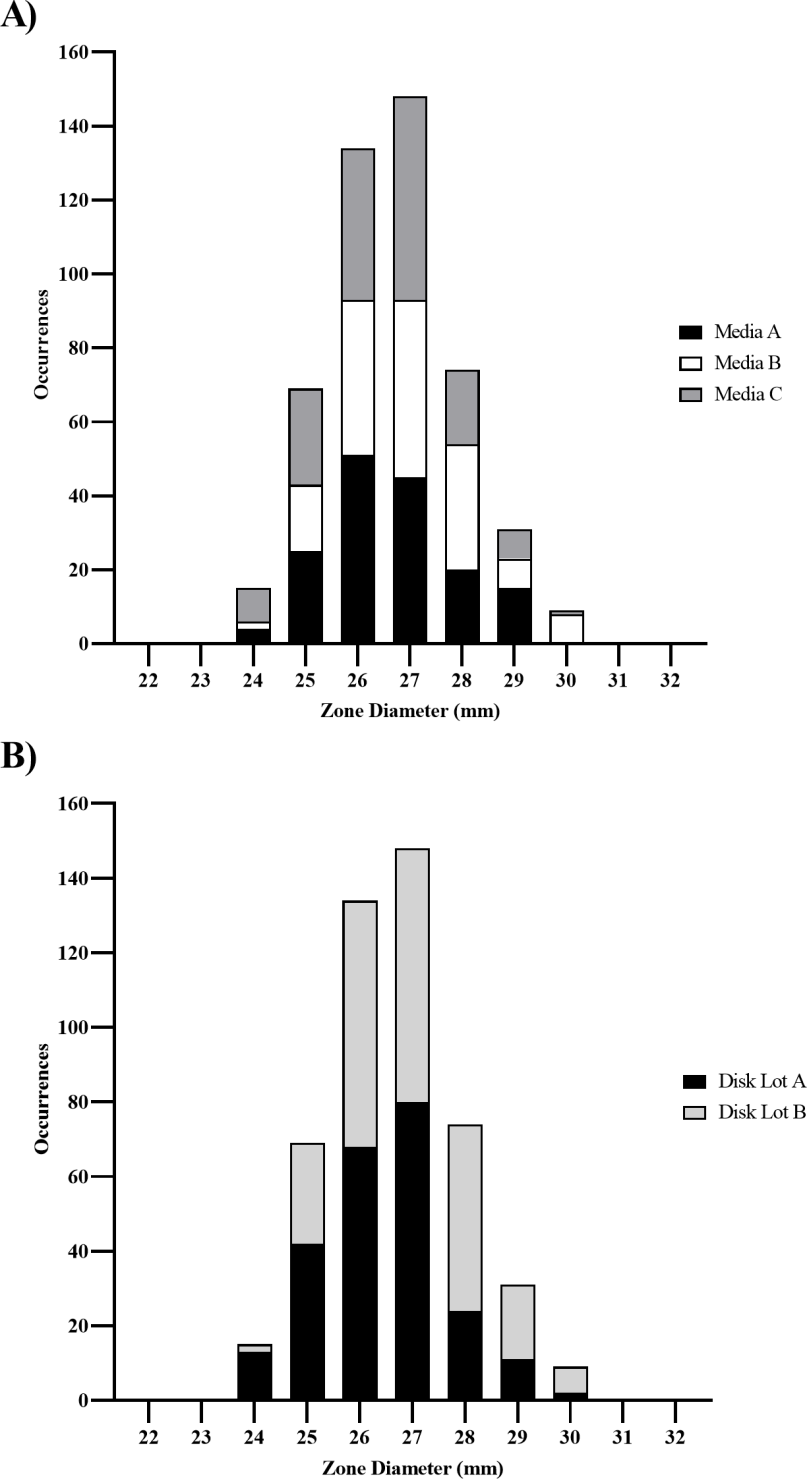
Frequency distribution of replicates of zone diameters with sulbactam/durlobactam 10/10 µg disk with *A. baumannii* NCTC 13304 from multi-lab study by media lot (**A**) and disk lot (**B**).

Similarly, a multi-lab study was completed to establish disk diffusion quality control ranges for sulbactam-durlobactam (10/10 µg) disks against *E. coli* ATCC 25922 and *A. baumannii* NCTC 13304 utilizing eight laboratories, three lots of media from different manufacturers, and two commercially prepared disk lots from different manufacturers according to CLSI guidelines (21). All of the replicates for the control disks ampicillin-sulbactam 10/10 µg and meropenem 10 µg disks tested with *E. coli* ATCC 25922 were within CLSI established quality control ranges (data not shown) (28). For the sulbactam-durlobactam (10/10 µg) disk against *E. coli* ATCC 25922, 99.8% of reported zone diameter results were within a 7 mm range of 26–32 mm as calculated by the Gavan statistic (Table 1); however, it should be noted that this growth inhibition primarily reflects the antibacterial activity of durlobactam against this strain and does not control for both components of the combination (4, 23). Replicates for zone diameter results with sulbactam-durlobactam (10/10 µg) disks for *A. baumannii* NCTC 13304 are shown in Fig. 6. The zone diameter distribution was unimodal with a mode of 27 mm. No bias was observed between the different media or disk lots. The Gavan statistic calculated a 5 mm QC range (25–29 mm) containing 95.0% (456/480) of sulbactam-durlobactam zone diameter values, whereas the RangeFinder statistical program calculated a 7 mm QC range (24–30 mm) containing 100.0% (480/480) of sulbactam-durlobactam (10/10 µg) zone diameter values (23, 24). No laboratories were determined to be statistical outliers for their mean, median, or modal sulbactam-durlobactam zone diameter values. These data were used by CLSI to approve a zone diameter quality control range of 24–30 mm with *A. baumannii* NCTC 13304 (Table 1) (28).

The results of these studies established antimicrobial susceptibility testing methods and quality control ranges for sulbactam-durlobactam using a systematic approach that assessed the test to correctly categorize isolates of *A. baumannii* that were predicted to be susceptible or resistant based on β-lactamase expression. MIC testing conducted with doubling dilutions of sulbactam in the presence of 4 µg/mL of durlobactam and disk diffusion tests containing 10 µg of both components were determined to be the best for meeting those criteria. Subsequently, quality control ranges for both MIC and disk tests were established and approved by CLSI. These studies will enable clinical laboratories to perform susceptibility tests with accurate and reproducible methods that will ensure the best care for patients.

## SUPPLEMENTAL MATERIAL

Table S1, Figures S1-S5 jcm.01228-23-s0001.docx Table shows MICs of isolates. Figures depict scattergrams of different disk contents.

ASM does not own the copyrights to Supplemental Material that may be linked to, or accessed through, an article. The authors have granted ASM a non-exclusive, world-wide license to publish the Supplemental Material files. Please contact the corresponding author directly for reuse.
