# Supplementary material for: Sulbactam-durlobactam susceptibility test method development and quality control ranges for MIC and disk diffusion tests
Source: J Clin Microbiol. 2023 Dec 14;62(1):e01228-23. doi: 10.1128/jcm.01228-23 (PMC10793306; doi:10.1128/jcm.01228-23)
Supplement: Table S1, Figures S1-S5 — Table shows MICs of isolates. Figures depict scattergrams of different disk contents. [file jcm.01228-23-s0001.docx]

**Supplemental Materials**

**Sulbactam-Durlobactam Susceptibility Test Method Development and Quality Control Ranges for MIC and Disk Diffusion Tests**

Sarah M. McLeod^1,^*, Nicole M. Carter^1^, Michael D. Huband^2^, Maria M. Traczewski^3^, Patricia A. Bradford^4^ and Alita A. Miller^1,^ⱡ

^1^ Entasis Therapeutics Inc., an affiliate of Innoviva Specialty Therapeutics, Inc., Waltham, MA, USA

^2^ JMI Labs, North Liberty, IA, USA

^3^ Clinical Microbiology Institute, Wilsonville, OR, USA

^4^ Antimicrobial Development Specialists, LLC, Nyack, NY, USA

ⱡ Author’s present affiliation: Design Therapeutics, Carlsbad, CA, USA

*Corresponding author:

Sarah M. McLeod

Innoviva Specialty Therapeutics, Inc., an affiliate of Entasis Therapeutics Inc.

35 Gatehouse Drive

Waltham, MA, USA

[Sarah.McLeod@istx.com](mailto:Sarah.McLeod@istx.com)

Table S 1: MIC values (in µg/mL) for combinations of sulbactam and durlobactam, plus comparator compounds minocycline (MIN) and imipenem (IPM) and durlobactam alone (DUR)

| *A. baumannii* Isolate | Whole genome sequencing results for β-lactamase genes and PBP3 alleles^1^ | Sulbactam + Durlobactam concentration  (µg/mL) | | | | Sulbactam : Durlobactam (ratio) | | | Comparators^2^ | | |
| --- | --- | --- | --- | --- | --- | --- | --- | --- | --- | --- | --- |
|  |  | 0 | 1 | 2 | 4 | 4:1 | 2:1 | 1:1 | DUR | IPM | MIN |
| ARC5078 | ADC-like | 1 | 0.25 | 0.25 | 0.125 | 0.5 | 0.5 | 0.5 | 16 | ≤0.03 | 0.25 |
| ARC3492 | ADC-52-like; TEM-1; OXA-24; OXA-132; PBP3 [A546T] | 8 | 0.5 | 0.5 | 0.25 | 2 | 2 | 1 | 16 | >32 | 2 |
| ARC2582 | ADC-79-like; OXA-100 [G159S] | 2 | 0.5 | 0.5 | 0.5 | 0.5 | 0.5 | 0.5 | 64 | 0.25 | 0.25 |
| ARC2597 | ADC-19 [K4N]; OXA-506 | 1 | 0.5 | 0.5 | 0.5 | 0.5 | 0.5 | 0.5 | 64 | 0.25 | 0.25 |
| ARC3486 | ADC-30; TEM-1; OXA-66; OXA-72 | 32 | 4 | 1 | 0.5 | 4 | 2 | 2 | 64 | >32 | 4 |
| ARC3489 | ADC-76; OXA-68; OXA-235-like | 4 | 0.5 | 0.5 | 0.5 | 2 | 1 | 0.5 | 32 | 4 | 1 |
| ARC3494 | ADC-6-like; OXA-65 | 2 | 0.5 | 0.5 | 0.5 | 2 | 1 | 1 | >64 | 0.125 | 0.25 |
| ARC3495 | ADC-30 [T341S]; OXA-24; OXA-109 | 32 | 1 | 1 | 0.5 | 4 | 2 | 1 | 64 | >32 | 2 |
| ARC5083 | ADC-30; OXA-23; OXA-66 | 16 | 0.5 | 0.5 | 0.5 | 1 | 1 | 0.5 | 32 | 16 | 8 |
| ARC5090 | ADC-44-like; OXA-332-like | 2 | 1 | 1 | 0.5 | 1 | 1 | 1 | 64 | 0.125 | 0.5 |
| CDC299 | ADC-26; PER-7; OXA-23; OXA-104 | 32 | 1 | 1 | 0.5 | 4 | 2 | 1 | 32 | 32 | 8 |
| ARC2058 | ADC-3-like; OXA-259 | 4 | 1 | 1 | 1 | 1 | 1 | 1 | >64 | 0.5 | 0.25 |
| ARC2674 | ADC-30; SHV-12; OXA-113 | 8 | 1 | 2 | 1 | 2 | 2 | 2 | >64 | 4 | 16 |
| ARC2681 | ADC-52-like; TEM-1; OXA-24; OXA-132 | 32 | 1 | 1 | 1 | 4 | 2 | 2 | >64 | 32 | 8 |
| ARC2682 | ADC-52-like; SHV-5; OXA-113 | 4 | 2 | 1 | 1 | 2 | 2 | 1 | >64 | 16 | 0.5 |
| ARC2777 | ADC-25-like; TEM-1; OXA-172 | 8 | 1 | 1 | 1 | 4 | 4 | 4 | >64 | 8 | 2 |
| ARC2778 | ADC-52-like [K28fs]; TEM-1; OXA-24; OXA-65 | 8 | 1 | 1 | 1 | 2 | 2 | 1 | >64 | >32 | 4 |
| ARC3484 | ADC-5; TEM-1; OXA-23; OXA-64 | 32 | 4 | 1 | 1 | 4 | 4 | 2 | 64 | >32 | 2 |
| ARC3488 | ADC-76; OXA-68; OXA-235-like | 8 | 1 | 1 | 1 | 2 | 2 | 1 | 64 | 4 | 1 |
| ARC3491 | ADC-like; OXA-215 | 1 | 1 | 1 | 1 | 0.5 | 0.5 | 0.5 | 16 | 0.25 | 0.5 |
| ARC3515 | ADC-26 [N341T]; OXA-58; OXA-64 | 8 | 2 | 1 | 1 | 4 | 2 | 2 | >64 | 16 | 2 |
| ARC3659 | ADC-79; OXA-23; OXA-69 | 8 | 1 | 1 | 1 | 2 | 1 | 1 | 64 | 8 | 1 |
| ARC5079 | ADC-52-like; OXA-65; OXA-72 | 32 | 1 | 1 | 1 | 4 | 4 | 2 | >64 | >32 | 1 |
| ARC5087 | ADC-30; OXA-23; OXA-66 | 64 | 1 | 1 | 1 | 2 | 2 | 1 | 64 | 32 | 8 |
| ARC5091 | ADC-25-like; OXA-23; OXA-82 | 8 | 1 | 1 | 1 | 2 | 1 | 1 | >64 | 32 | 4 |
| ARC5092 | ADC-5-like; OXA-23; OXA-64 | 8 | 1 | 1 | 1 | 2 | 2 | 1 | 64 | 32 | 8 |
| ATCC 19606 | ADC-2 [N260H]; OXA-98 | 1 | 1 | 1 | 1 | 2 | 2 | 2 | 64 | 0.5 | 0.5 |
| CDC281 | ADC-30; TEM-1; OXA-82 | 32 | 2 | 2 | 1 | 2 | 2 | 2 | 64 | 4 | 8 |
| ARC2635 | ADC-52-like; TEM-1; OXA-24; OXA-65 | 32 | 4 | 2 | 2 | 8 | 4 | 2 | 32 | >32 | 1 |
| ARC2720 | ADC-18; OXA-500 | 4 | 2 | 2 | 2 | 2 | 1 | 1 | >64 | 0.5 | 0.5 |
| ARC2782 | ADC-79; PER-1; TEM-1; OXA-23; OXA-66 | 32 | 2 | 2 | 2 | 4 | 2 | 1 | 64 | 32 | 4 |
| ARC3493 | ADC-1; OXA-24; OXA-66; PBP3 [I592N] | 64 | 2 | 2 | 2 | 4 | 2 | 2 | 64 | >32 | 2 |
| ARC3513 | ADC-5; TEM-1; OXA-23; OXA-65 | 16 | 2 | 2 | 2 | 4 | 4 | 2 | >64 | 32 | 1 |
| ARC3658 | ADC-25; PER-1; TEM-1; OXA-23; OXA-66 | 64 | 2 | 2 | 2 | 4 | 4 | 2 | >64 | 32 | 4 |
| ARC5076 | ADC-30 [A245E]; TEM-1; OXA-23; OXA-66 | 32 | 2 | 2 | 2 | 4 | 4 | 4 | >64 | >32 | 16 |
| ARC5086 | ADC-30; TEM-1; OXA-66; OXA-72; OXA-398 | 32 | 4 | 4 | 2 | 8 | 8 | 8 | >64 | >32 | 16 |
| ARC5088 | ADC-38-like; OXA-20; OXA-58; OXA-66 | 8 | 2 | 2 | 2 | 4 | 4 | 4 | >64 | 32 | 2 |
| ARC5089 | ADC-11; PER-1 [V236G]; TEM-1; OXA-23; OXA-66 | 32 | 2 | 2 | 2 | 8 | 4 | 2 | >64 | 32 | 0.5 |
| CDC277 | ADC-52-like; TEM-1; OXA-24; OXA-65 | 32 | 8 | 4 | 2 | 4 | 4 | 4 | >64 | 64 | 2 |
| CDC286 | ADC-30; OXA-24; OXA-66 | 32 | 4 | 4 | 2 | 8 | 4 | 4 | 64 | >64 | 8 |
| CDC287 | ADC-30; OXA-66; OXA-72 | 32 | 16 | 4 | 2 | 8 | 4 | 2 | 64 | >64 | 8 |
| CDC303 | ADC-30 [A245E]; OXA-23; OXA-66 | 64 | >64 | >64 | 2 | 64 | 32 | 32 | >64 | 32 | 16 |
| CDC305 | ADC-52-like; TEM-1; OXA-24; OXA-65 | 8 | 4 | 2 | 2 | 4 | 2 | 2 | >64 | >64 | 1 |
| CDC311 | ADC-56; OXA-23; OXA-82 | 32 | 4 | 4 | 2 | 8 | 4 | 4 | >64 | 64 | 4 |
| NCTC 13304 | ADC-30; TEM-1: OXA-23; OXA-66 | 32 | 8 | 4 | 2 | 8 | 8 | 8 | 64 | 64 | 4 |
| ARC2636 | ADC-52-like; TEM-1; OXA-24; OXA-65 | 32 | 4 | 4 | 4 | 8 | 4 | 2 | >64 | >32 | 2 |
| ARC2675 | ADC-25-like; SHV-5; OXA-113 | 16 | 4 | 4 | 4 | 8 | 4 | 4 | >64 | >32 | 1 |
| ARC3485 | ADC-25-like; OXA-82 | 8 | 4 | 4 | 4 | 2 | 2 | 2 | >64 | 16 | 2 |
| ARC5073 | ADC-26; PER-1; TEM-1; OXA-23; OXA-64 | 32 | 4 | 4 | 4 | 4 | 4 | 4 | >64 | 32 | 4 |
| ARC5075 | ADC-25; SHV-5; OXA-113 | 64 | 4 | 4 | 4 | 16 | 8 | 8 | >64 | 32 | 2 |
| ARC5077 | ADC-82; OXA-66 [K42*]; OXA-72 | 32 | 4 | 4 | 4 | 8 | 8 | 8 | >64 | >32 | 16 |
| ARC5080 | ADC-7-like; OXA-24; OXA-71; PBP3 [G523V] | 64 | 4 | 4 | 4 | 8 | 8 | 4 | >64 | >32 | 1 |
| ARC5081 | ADC-80; ADC-81-like; OXA-23; OXA-94 | 16 | 8 | 4 | 4 | 4 | 4 | 4 | >64 | 16 | 0.25 |
| ARC5082 | ADC-30 [A245E]; OXA-23; OXA-66 | 64 | 16 | 8 | 4 | 8 | 8 | 8 | >64 | >32 | 8 |
| ARC5957 | ADC-73; TEM-1; OXA-23; OXA-66 | 32 | 4 | 4 | 4 | 4 | 4 | 4 | >64 | >32 | 8 |
| CDC280 | ADC-30; TEM-1; OXA-66 | >64 | 8 | 4 | 4 | 16 | 16 | 16 | >64 | 1 | 16 |
| CDC283 | ADC-30 [A245E]; TEM-1; OXA-23; OXA-66 | 32 | 16 | 8 | 4 | 16 | 16 | 8 | >64 | 32 | 16 |
| CDC284 | ADC-52 [K28fs]; TEM-1; OXA-24; OXA-65 | 32 | 8 | 4 | 4 | 8 | 8 | 4 | >64 | 64 | 1 |
| CDC285 | ADC-52-like; TEM-1; OXA-24; OXA-65 | 64 | 16 | 8 | 4 | 16 | 16 | 4 | 64 | >64 | 1 |
| CDC297 | ADC-30 [A245E]; OXA-23; OXA-66 | 32 | 32 | 8 | 4 | 8 | 8 | 8 | 64 | 32 | 16 |
| CDC300 | ADC-30; OXA-66 | 32 | 4 | 4 | 4 | 4 | 4 | 4 | >64 | 2 | 8 |
| CDC304 | ADC-30; OXA-66; OXA-72 | 32 | 8 | 4 | 4 | 8 | 8 | 8 | 32 | 32 | 32 |
| CDC306 | ADC-52-like; TEM-1; OXA-24; OXA-65 | >64 | 4 | 4 | 4 | 8 | 4 | 4 | >64 | >64 | 4 |
| CDC307 | ADC-30; OXA-66; OXA-237 | 16 | 8 | 8 | 4 | 16 | 16 | 8 | >64 | 8 | 16 |
| CDC308 | ADC-30; TEM-1; OXA-113 | 32 | 8 | 4 | 4 | 8 | 4 | 4 | >64 | 8 | 16 |
| ARC2059 | ADC-56 [T341S]; CARB-2; OXA-69; PBP3 [T526S] | 8 | 8 | 8 | 8 | 8 | 8 | 8 | >64 | 0.5 | 2 |
| ARC3487 | ADC-38-like; OXA-20; OXA-58; OXA-66; PBP3 [A346V; H370Y] | 8 | 8 | 8 | 8 | 8 | 8 | 8 | >64 | 16 | 2 |
| ARC3657 | ADC-39-like; OXA-130; PBP3 [T511S] | 32 | 8 | 8 | 8 | 8 | 8 | 8 | >64 | 1 | 0.5 |
| ARC5950 | ADC-11; OXA-23; OXA-69; PBP3 [T526S] | 64 | 8 | 8 | 8 | 16 | 16 | 16 | >64 | 32 | 8 |
| ARC6304 | ADC-30; OXA-23; OXA-66; PBP3 [T526S] | 64 | 16 | 8 | 8 | 16 | 16 | 8 | >64 | >32 | 16 |
| CDC278 | ADC-30 [A245E]; TEM-1; OXA-23; OXA-66 | 32 | 32 | 32 | 8 | 16 | 16 | 16 | >64 | 64 | 16 |
| ARC5085 | ADC-10-like; TEM-1; OXA-407 [L167V]; PBP3 [T526S] | 16 | 16 | 16 | 16 | 16 | 16 | 16 | >64 | 8 | 32 |
| ARC5580 | ADC-91; OXA-23; OXA-68; PBP3 [T526S] | 32 | 16 | 16 | 16 | 16 | 16 | 16 | >64 | 32 | 1 |
| ARC5980 | ADC-79-like; OXA-23; OXA-69; PBP3 [T526S] | 32 | 32 | 16 | 16 | 16 | 16 | 16 | >64 | 32 | 0.25 |
| ARC6651 | ADC-73; OXA-2; OXA-66; PBP3 [A515V] | 64 | 16 | 16 | 16 | 32 | 16 | 16 | >64 | 64 | ND |
| ARC5615 | ADC-2 [N260H]; OXA-98; OXA-235; NDM-1 | >64 | 32 | 32 | 32 | 32 | 32 | 32 | >64 | >32 | 0.5 |
| ARC6650 | ADC-25; OXA-23; OXA-66; PBP3 [T526S] | 64 | 32 | 32 | 32 | 32 | 32 | 32 | >64 | 32 | ND |
| CDC279 | ADC-30 [A245E]; TEM-1; OXA-23; OXA-66 | 32 | 64 | 64 | 32 | 64 | 64 | 32 | >64 | 64 | 16 |
| CDC296 | ADC-30; OXA-23; OXA-223 | 32 | 64 | 32 | 32 | 32 | 16 | 16 | >64 | 64 | 2 |
| CDC298 | ADC-30; TEM-1; OXA-66; OXA-237 | >64 | >64 | 64 | 32 | 64 | 32 | 32 | >64 | 16 | 8 |
| ARC6197 | ADC-26; TEM-1; OXA-64; NDM-1 | >64 | 64 | 64 | 64 | 64 | 32 | 32 | >64 | >32 | 2 |
| ARC6645 | CARB-2; OXA-58; OXA-9; NDM-1 | >64 | >64 | 64 | 64 | 64 | 64 | 32 | >64 | >64 | ND |
| ARC6647 | ADC-26; OXA-64; PER-7; NDM-1 | >64 | 64 | 64 | 64 | 64 | 64 | 32 | 64 | >64 | ND |
| ARC6646 | ADC-30; OXA-23; OXA-66; PBP3 [T526S] | >64 | 64 | 64 | 64 | 64 | 64 | 64 | >64 | 32 | ND |
| CDC282 | ADC-30 [A245E]; TEM-1; OXA-23; OXA-66 | >64 | >64 | >64 | 64 | 64 | 64 | 32 | >64 | 64 | 16 |
| ARC3882 | ADC-79-like; OXA-23; OXA-371; NDM-1; PBP3 [T526S] | >64 | >64 | >64 | >64 | >64 | >64 | >64 | >64 | >32 | 0.5 |
| ARC6652 | OXA-94; NDM-1; subclass B3 metallo-β-lactamase | >64 | >64 | >64 | >64 | >64 | >64 | 64 | >64 | >64 | ND |
| ARC6644 | ADC-30; TEM-1; OXA-83; PBP3 [A515V] | 64 | >64 | >64 | >64 | >64 | >64 | >64 | >64 | 16 | ND |
| ARC6653 | ADC-82; OXA-23; OXA-66; PBP3 [T526S] | 64 | >64 | >64 | >64 | >64 | >64 | >64 | >64 | >64 | ND |
| ARC6489 | ADC-26; OXA-64; NDM-1 | >64 | >64 | >64 | >64 | >64 | 64 | 64 | ND | >64 | ND |
| ARC6182* | ATCC 17978 PBP3 S390T | >64 | >64 | >64 | >64 | >64 | >64 | 64 | >64 | 0.125 | ND |

^1^Isolates encoding PBP3 mutations predicted to confer resistance to sulbactam or β-lactamases not inhibited by durlobactam are shaded in grey. ^2^DUR = durlobactam; IPM = imipenem; MIN = minocycline; ND = not determined. *Laboratory-generated mutant resistant to sulbactam (Penwell WF et al. Molecular mechanisms of sulbactam antibacterial activity and resistance determinants in *Acinetobacter baumannii*. Antimicrob Agents Chemother. 2015 Mar;59(3):1680-9).

**Figure S1:** Scattergram and tabular presentation of error rates, based on the CLSI error-rate bounded method when comparing sulbactam-durlobactam (fixed 4 µg/mL) MIC results to sulbactam/durlobactam (10/10 µg disks) zone diameter values in duplicate testing against 58 *Acinetobacter baumannii-calcoaceticus* species complex isolates

| **Sulbactam-Durlobactam (fixed 4 µg/mL)** | I+3 | >32 | 2 | 1 | 1 |  |  |  |  |  |  |  |  |  |  |  |  |  |  |  |  |  |  |  |  |  |  |  |  |  |  |  |  |  |
| --- | --- | --- | --- | --- | --- | --- | --- | --- | --- | --- | --- | --- | --- | --- | --- | --- | --- | --- | --- | --- | --- | --- | --- | --- | --- | --- | --- | --- | --- | --- | --- | --- | --- | --- |
|  | I+2 | 32 |  |  |  |  |  |  |  |  |  |  |  |  |  |  |  |  |  |  |  |  |  |  |  |  |  |  |  |  |  |  |  |  |
|  | I+1 | 16 | 2 |  |  |  |  | 1 | 3 |  |  |  |  |  |  |  |  |  |  |  |  |  |  |  |  |  |  |  |  |  |  |  |  |  |
|  | I | 8 |  |  | 2 |  |  |  | 2 |  | 2 |  |  |  | 1 | 1 |  |  |  |  |  |  |  |  |  |  |  |  |  |  |  |  |  |  |
|  | I-1 | 4 |  |  |  |  |  |  |  |  |  |  |  |  | 2 | 2 |  |  |  |  |  |  |  |  |  |  |  |  |  |  |  |  |  |  |
|  | I-2 | 2 |  |  |  |  |  |  |  |  |  |  |  |  |  | 2 |  | 7 | 5 | 3 | 1 |  |  |  |  |  |  |  |  |  |  |  |  |  |
|  | I-3 | 1 |  |  |  |  |  |  |  |  |  |  |  |  |  |  | 2 |  | 2 | 7 | 1 | 2 | 4 | 1 | 1 |  |  |  |  |  |  |  |  |  |
|  | I-4 | 0.5 |  |  |  |  |  |  |  |  |  |  |  |  |  |  |  |  |  |  | 2 | 6 | 6 | 5 | 7 | 6 | 2 |  |  |  |  |  |  |  |
|  | I-5 | 0.25 |  |  |  |  |  |  |  |  |  |  |  |  |  |  |  |  |  |  |  |  | 2 |  | 2 |  |  | 1 | 2 | 1 |  |  |  |  |
|  | I-6 | 0.12 |  |  |  |  |  |  |  |  |  |  |  |  |  |  |  |  |  |  |  |  |  | 2 |  | 2 |  |  | 4 |  |  |  | 1 | 1 |
|  | I-7 | 0.06 |  |  |  |  |  |  |  |  |  |  |  |  |  |  |  |  |  |  |  |  |  |  |  |  |  |  |  |  |  |  |  |  |
|  | I-8 | 0.03 |  |  |  |  |  |  |  |  |  |  |  |  |  |  |  |  |  |  |  |  |  |  |  |  |  |  |  |  |  |  |  |  |
|  | I-9 | ≤0.015 |  |  |  |  |  |  |  |  |  |  |  |  |  |  |  |  |  |  |  |  |  |  |  | 1 | 3 |  |  |  |  |  |  |  |
|  |  |  | 6 | 7 | 8 | 9 | 10 | 11 | 12 | 13 | 14 | 15 | 16 | 17 | 18 | 19 | 20 | 21 | 22 | 23 | 24 | 25 | 26 | 27 | 28 | 29 | 30 | 31 | 32 | 33 | 34 | 35 | 36 | 37 |

**Sulbactam/Durlobactam 10/10 µg Disk (mm)**

**Scattergram and error rate table for the optimal calculated sulbactam/durlobactam (10/10 µg) disk breakpoints of ≥18.0 (S) and ≤13.0 (R)**

| **MIC Range** | **Number** | **Very Major Errors**  **n (%)** | **Major Errors**  **n (%)** | **Minor Errors**  **n (%)** |
| --- | --- | --- | --- | --- |
| ≥I+2 | 4 | 0 | N/A | 0 |
| I+1 to I-1 | 18 | 0 | 0 | 6 (33.3) |
| ≤I-2 | 94 | N/A | 0 | 0 |
| **Total** | **116** | **0** | **0** | **6 (5.2)** |

N/A = not applicable

**Figure S2:** Scattergram and tabular presentation of error rates, based on the CLSI error-rate bounded method when comparing sulbactam-durlobactam (fixed 4 µg/mL) MIC results to sulbactam/durlobactam (10/5 µg disks) zone diameter values in duplicate testing against 58 *Acinetobacter baumannii calcoaceticus* species complex isolates

| **Sulbactam-Durlobactam MIC (µg/mL)** | I+3 | >32 | 2 | 2 |  |  |  |  |  |  |  |  |  |  |  |  |  |  |  |  |  |  |  |  |  |  |  |  |  |  |  |  |  |  |
| --- | --- | --- | --- | --- | --- | --- | --- | --- | --- | --- | --- | --- | --- | --- | --- | --- | --- | --- | --- | --- | --- | --- | --- | --- | --- | --- | --- | --- | --- | --- | --- | --- | --- | --- |
|  | I+2 | 32 |  |  |  |  |  |  |  |  |  |  |  |  |  |  |  |  |  |  |  |  |  |  |  |  |  |  |  |  |  |  |  |  |
|  | I+1 | 16 | 2 |  |  |  | 2 | 1 | 1 |  |  |  |  |  |  |  |  |  |  |  |  |  |  |  |  |  |  |  |  |  |  |  |  |  |
|  | I | 8 |  |  |  |  | 2 | 2 |  | 1 | 1 |  | 1^a^ | 1^a^ |  |  |  |  |  |  |  |  |  |  |  |  |  |  |  |  |  |  |  |  |
|  | I-1 | 4 |  |  |  |  |  |  |  |  |  |  |  | 2 | 2 |  |  |  |  |  |  |  |  |  |  |  |  |  |  |  |  |  |  |  |
|  | I-2 | 2 |  |  |  |  |  |  |  |  |  |  |  | 1 | 1 | 2 | 7 | 5 |  | 2 |  |  |  |  |  |  |  |  |  |  |  |  |  |  |
|  | I-3 | 1 |  |  |  |  |  |  |  |  |  |  |  |  |  | 2 |  | 3 | 7 | 2 |  | 4 | 2 |  |  |  |  |  |  |  |  |  |  |  |
|  | I-4 | 0.5 |  |  |  |  |  |  |  |  |  |  |  |  |  |  |  |  | 2 | 4 | 1 | 9 | 4 | 6 | 6 | 2 |  |  |  |  |  |  |  |  |
|  | I-5 | 0.25 |  |  |  |  |  |  |  |  |  |  |  |  |  |  |  |  |  |  |  | 2 |  | 2 |  |  | 1 | 2 | 1 |  |  |  |  |  |
|  | I-6 | 0.12 |  |  |  |  |  |  |  |  |  |  |  |  |  |  |  |  |  |  |  |  | 2 | 1 | 1 |  |  | 3 | 1 | 1 |  | 1 |  |  |
|  | I-7 | 0.06 |  |  |  |  |  |  |  |  |  |  |  |  |  |  |  |  |  |  |  |  |  |  |  |  |  |  |  |  |  |  |  |  |
|  | I-8 | 0.03 |  |  |  |  |  |  |  |  |  |  |  |  |  |  |  |  |  |  |  |  |  |  |  |  |  |  |  |  |  |  |  |  |
|  | I-9 | ≤0.015 |  |  |  |  |  |  |  |  |  |  |  |  |  |  |  |  |  |  |  |  |  |  | 2 | 2 |  |  |  |  |  |  |  |  |
|  |  |  | 6 | 7 | 8 | 9 | 10 | 11 | 12 | 13 | 14 | 15 | 16 | 17 | 18 | 19 | 20 | 21 | 22 | 23 | 24 | 25 | 26 | 27 | 28 | 29 | 30 | 31 | 32 | 33 | 34 | 35 | 36 | 37 |

**Sulbactam/Durlobactam 10/5 µg Disk (mm)**

^a^ JMI collection number 845887

**Scattergram and error rate table for the optimal calculated sulbactam/durlobactam (10/5 µg) disk breakpoints of ≥17.0 (S) and ≤12.0 (R)**

| **MIC Range** | **Number** | **Very Major Errors**  **n (%)** | **Major Errors**  **n (%)** | **Minor Errors**  **n (%)** |
| --- | --- | --- | --- | --- |
| ≥I+2 | 4 | 0 | N/A | 0 |
| I+1 to I-1 | 18 | 0 | 0 | 5 (27.8) |
| ≤I-2 | 94 | N/A | 0 | 0 |
| **Total** | **116** | **0** | **0** | **5 (4.3)** |

N/A = not applicable

**Figure S3:** Scattergram and tabular presentation of error rates, based on the CLSI error-rate bounded method when comparing sulbactam-durlobactam (fixed 4 µg/mL) MIC results to sulbactam/durlobactam (5/10 µg disks) zone diameter values in duplicate testing against 58 *Acinetobacter baumannii calcoaceticus* species complex isolates.

| **Sulbactam-Durlobactam MIC (µg/mL)** | I+3 | >32 | 2 | 2 |  |  |  |  |  |  |  |  |  |  |  |  |  |  |  |  |  |  |  |  |  |  |  |  |  |  |  |  |  |  |
| --- | --- | --- | --- | --- | --- | --- | --- | --- | --- | --- | --- | --- | --- | --- | --- | --- | --- | --- | --- | --- | --- | --- | --- | --- | --- | --- | --- | --- | --- | --- | --- | --- | --- | --- |
|  | I+2 | 32 |  |  |  |  |  |  |  |  |  |  |  |  |  |  |  |  |  |  |  |  |  |  |  |  |  |  |  |  |  |  |  |  |
|  | I+1 | 16 | 6 |  |  |  |  |  |  |  |  |  |  |  |  |  |  |  |  |  |  |  |  |  |  |  |  |  |  |  |  |  |  |  |
|  | I | 8 | 4 |  |  | 2 |  |  |  |  | 1 | 1 |  |  |  |  |  |  |  |  |  |  |  |  |  |  |  |  |  |  |  |  |  |  |
|  | I-1 | 4 |  |  |  |  |  |  |  | 1 |  | 3 |  |  |  |  |  |  |  |  |  |  |  |  |  |  |  |  |  |  |  |  |  |  |
|  | I-2 | 2 |  |  |  |  |  |  |  |  |  | 3 |  | 2 | 6 | 2 | 3 | 2 |  |  |  |  |  |  |  |  |  |  |  |  |  |  |  |  |
|  | I-3 | 1 |  |  |  |  |  |  |  |  |  |  |  |  |  | 3 | 4 | 4 | 2 | 3 | 2 | 1 | 1 |  |  |  |  |  |  |  |  |  |  |  |
|  | I-4 | 0.5 |  |  |  |  |  |  |  |  |  |  |  |  |  |  |  |  |  | 8 | 3 | 7 | 7 | 3 | 6 |  |  |  |  |  |  |  |  |  |
|  | I-5 | 0.25 |  |  |  |  |  |  |  |  |  |  |  |  |  |  |  |  |  |  |  | 2 | 1 | 1 |  |  | 2 | 2 |  |  |  |  |  |  |
|  | I-6 | 0.12 |  |  |  |  |  |  |  |  |  |  |  |  |  |  |  |  |  |  |  | 2 |  | 1 | 1 | 4 |  |  | 1 | 1 |  |  |  |  |
|  | I-7 | 0.06 |  |  |  |  |  |  |  |  |  |  |  |  |  |  |  |  |  |  |  |  |  |  |  |  |  |  |  |  |  |  |  |  |
|  | I-8 | 0.03 |  |  |  |  |  |  |  |  |  |  |  |  |  |  |  |  |  |  |  |  |  |  |  |  |  |  |  |  |  |  |  |  |
|  | I-9 | ≤0.015 |  |  |  |  |  |  |  |  |  |  |  |  |  |  |  |  |  |  |  |  |  | 1 | 3 |  |  |  |  |  |  |  |  |  |
|  |  |  | 6 | 7 | 8 | 9 | 10 | 11 | 12 | 13 | 14 | 15 | 16 | 17 | 18 | 19 | 20 | 21 | 22 | 23 | 24 | 25 | 26 | 27 | 28 | 29 | 30 | 31 | 32 | 33 | 34 | 35 | 36 | 37 |

**Sulbactam/Durlobactam 5/10 µg Disk (mm)**

**Scattergram and error rate table for the optimal calculated sulbactam/durlobactam (5/10 µg) disk breakpoints of ≥13.0 (S) and ≤8.0 (R)**

| **MIC Range** | **Number** | **Very Major Errors**  **n (%)** | **Major Errors**  **n (%)** | **Minor Errors**  **n (%)** |
| --- | --- | --- | --- | --- |
| ≥I+2 | 4 | 0 | N/A | 0 |
| I+1 to I-1 | 18 | 0 | 0 | 6 (33.3) |
| ≤I-2 | 94 | N/A | 0 | 0 |
| **Total** | **116** | **0** | **0** | **6 (5.2)** |

N/A = not applicable

**Figure S4:** Scattergram and tabular presentation of error rates, based on the CLSI error-rate bounded method when comparing sulbactam-durlobactam (fixed 4 µg/mL) MIC results to sulbactam/durlobactam (5/5 µg disks) zone diameter values in duplicate testing against 58 *Acinetobacter baumannii calcoaceticus* species complex isolates

| **Sulbactam-Durlobactam MIC (µg/mL)** | I+3 | >32 | 2 | 2 |  |  |  |  |  |  |  |  |  |  |  |  |  |  |  |  |  |  |  |  |  |  |  |  |  |  |  |  |  |  |  |  |  |
| --- | --- | --- | --- | --- | --- | --- | --- | --- | --- | --- | --- | --- | --- | --- | --- | --- | --- | --- | --- | --- | --- | --- | --- | --- | --- | --- | --- | --- | --- | --- | --- | --- | --- | --- | --- | --- | --- |
|  | I+2 | 32 |  |  |  |  |  |  |  |  |  |  |  |  |  |  |  |  |  |  |  |  |  |  |  |  |  |  |  |  |  |  |  |  |  |  |  |
|  | I+1 | 16 | 6 |  |  |  |  |  |  |  |  |  |  |  |  |  |  |  |  |  |  |  |  |  |  |  |  |  |  |  |  |  |  |  |  |  |  |
|  | I | 8 | 6 |  |  |  |  |  |  | 1^a^ | 1^a^ |  |  |  |  |  |  |  |  |  |  |  |  |  |  |  |  |  |  |  |  |  |  |  |  |  |  |
|  | I-1 | 4 |  |  |  |  |  |  |  | 1 | 1 | 2 |  |  |  |  |  |  |  |  |  |  |  |  |  |  |  |  |  |  |  |  |  |  |  |  |  |
|  | I-2 | 2 |  |  |  |  |  |  |  |  | 1 | 2 |  | 6 | 2 | 4 | 1 | 2 |  |  |  |  |  |  |  |  |  |  |  |  |  |  |  |  |  |  |  |
|  | I-3 | 1 |  |  |  |  |  |  |  |  |  |  |  |  | 2 | 2 | 3 | 6 | 2 | 2 |  | 3 |  |  |  |  |  |  |  |  |  |  |  |  |  |  |  |
|  | I-4 | 0.5 |  |  |  |  |  |  |  |  |  |  |  |  |  |  | 1 | 1 | 3 | 7 | 3 | 5 | 6 | 3 | 5 |  |  |  |  |  |  |  |  |  |  |  |  |
|  | I-5 | 0.25 |  |  |  |  |  |  |  |  |  |  |  |  |  |  |  |  | 1 |  | 1 |  | 2 |  | 1 | 2 | 1 |  |  |  |  |  |  |  |  |  |  |
|  | I-6 | 0.12 |  |  |  |  |  |  |  |  |  |  |  |  |  |  |  |  |  |  |  | 2 | 2 |  |  | 4 |  |  | 1 | 1 |  |  |  |  |  |  |  |
|  | I-7 | 0.06 |  |  |  |  |  |  |  |  |  |  |  |  |  |  |  |  |  |  |  |  |  |  |  |  |  |  |  |  |  |  |  |  |  |  |  |
|  | I-8 | 0.03 |  |  |  |  |  |  |  |  |  |  |  |  |  |  |  |  |  |  |  |  |  |  |  |  |  |  |  |  |  |  |  |  |  |  |  |
|  | I-9 | ≤0.015 |  |  |  |  |  |  |  |  |  |  |  |  |  |  |  |  |  |  |  |  |  | 4 |  |  |  |  |  |  |  |  |  |  |  |  |  |
|  |  |  | 6 | 7 | 8 | 9 | 10 | 11 | 12 | 13 | 14 | 15 | 16 | 17 | 18 | 19 | 20 | 21 | 22 | 23 | 24 | 25 | 26 | 27 | 28 | 29 | 30 | 31 | 32 | 33 | 34 | 35 | 36 | 37 | 38 | 39 | 40 |

**Sulbactam/Durlobactam 5/5 µg Disk (mm)**

^a^ JMI collection number 845887

**Scattergram and error rate table for the optimal calculated sulbactam/durlobactam (5/5 µg) disk breakpoints of ≥14.0 (S) and ≤10.0 (R)**

| **MIC Range** | **Number** | **Very Major Errors**  **n (%)** | **Major Errors**  **n (%)** | **Minor Errors**  **n (%)** |
| --- | --- | --- | --- | --- |
| ≥I+2 | 4 | 0 | N/A | 0 |
| I+1 to I-1 | 18 | 0 | 0 | 8 (44.4) |
| ≤I-2 | 94 | N/A | 0 | 0 |
| **Total** | **116** | **0** | **0** | **8 (6.9)** |

**Figure S5:** Scattergram and tabular presentation of error rates, based on the CLSI error-rate bounded method when comparing sulbactam-durlobactam (fixed 4 µg/mL) MIC results to sulbactam/durlobactam (7.5/7.5 µg disks) zone diameter values in duplicate testing against 58 *Acinetobacter baumannii calcoaceticus* species complex isolates

| **Sulbactam-Durlobactam MIC (µg/mL)** | I+3 | >32 | 2 | 2 |  |  |  |  |  |  |  |  |  |  |  |  |  |  |  |  |  |  |  |  |  |  |  |  |  |  |  |  |  |  |  |  |  |
| --- | --- | --- | --- | --- | --- | --- | --- | --- | --- | --- | --- | --- | --- | --- | --- | --- | --- | --- | --- | --- | --- | --- | --- | --- | --- | --- | --- | --- | --- | --- | --- | --- | --- | --- | --- | --- | --- |
|  | I+2 | 32 |  |  |  |  |  |  |  |  |  |  |  |  |  |  |  |  |  |  |  |  |  |  |  |  |  |  |  |  |  |  |  |  |  |  |  |
|  | I+1 | 16 | 3 |  | 1 |  | 2 |  |  |  |  |  |  |  |  |  |  |  |  |  |  |  |  |  |  |  |  |  |  |  |  |  |  |  |  |  |  |
|  | I | 8 | 2 |  |  |  | 1^a^ | 3^a^ |  |  |  |  | 2 |  |  |  |  |  |  |  |  |  |  |  |  |  |  |  |  |  |  |  |  |  |  |  |  |
|  | I-1 | 4 |  |  |  |  |  |  |  |  |  |  | 2 | 2 |  |  |  |  |  |  |  |  |  |  |  |  |  |  |  |  |  |  |  |  |  |  |  |
|  | I-2 | 2 |  |  |  |  |  |  |  |  |  |  | 1 |  | 1 | 4 | 5 | 5 | 1 | 1 |  |  |  |  |  |  |  |  |  |  |  |  |  |  |  |  |  |
|  | I-3 | 1 |  |  |  |  |  |  |  |  |  |  |  |  |  | 2 |  | 3 | 8 | 1 | 2 | 2 | 2 |  |  |  |  |  |  |  |  |  |  |  |  |  |  |
|  | I-4 | 0.5 |  |  |  |  |  |  |  |  |  |  |  |  |  |  |  |  |  | 6 | 3 | 5 | 8 | 4 | 7 |  | 1 |  |  |  |  |  |  |  |  |  |  |
|  | I-5 | 0.25 |  |  |  |  |  |  |  |  |  |  |  |  |  |  |  |  |  |  |  | 2 |  | 2 |  |  | 2 | 2 |  |  |  |  |  |  |  |  |  |
|  | I-6 | 0.12 |  |  |  |  |  |  |  |  |  |  |  |  |  |  |  |  |  |  |  | 2 |  | 1 | 1 | 1 | 1 | 2 |  | 2 |  |  |  |  |  |  |  |
|  | I-7 | 0.06 |  |  |  |  |  |  |  |  |  |  |  |  |  |  |  |  |  |  |  |  |  |  |  |  |  |  |  |  |  |  |  |  |  |  |  |
|  | I-8 | 0.03 |  |  |  |  |  |  |  |  |  |  |  |  |  |  |  |  |  |  |  |  |  |  |  |  |  |  |  |  |  |  |  |  |  |  |  |
|  | I-9 | ≤0.015 |  |  |  |  |  |  |  |  |  |  |  |  |  |  |  |  |  |  |  |  |  |  | 2 | 2 |  |  |  |  |  |  |  |  |  |  |  |
|  |  |  | 6 | 7 | 8 | 9 | 10 | 11 | 12 | 13 | 14 | 15 | 16 | 17 | 18 | 19 | 20 | 21 | 22 | 23 | 24 | 25 | 26 | 27 | 28 | 29 | 30 | 31 | 32 | 33 | 34 | 35 | 36 | 37 | 38 | 39 | 40 |

**Sulbactam/Durlobactam 7.5/7.5 µg Disk (mm)**

^a^ JMI collection number 920570

**Scattergram and error rate table for the optimal calculated sulbactam/durlobactam (7.5/7.5 µg) disk breakpoints of ≥15.0 (S) and ≤10.0 (R)**

| **MIC Range** | **Number** | **Very Major Errors**  **n (%)** | | **Major Errors**  **n (%)** | | **Minor Errors**  **n (%)** | |
| --- | --- | --- | --- | --- | --- | --- | --- |
| ≥I+2 | 4 | | 0 | | N/A | | 0 |
| I+1 to I-1 | 18 | | 0 | | 0 | | 5 (27.8) |
| ≤I-2 | 94 | | N/A | | 0 | | 0 |
| **Total** | **116** | | **0** | | **0** | | **5 (4.3)** |

N/A = not applicable
